# NMR Spectroscopy in Glass Science: A Review of the Elements

**DOI:** 10.3390/ma11040476

**Published:** 2018-03-22

**Authors:** Randall Youngman

**Affiliations:** Science & Technology Division, Corning Incorporated, SP-AR-02-4, Corning, NY 14831, USA; youngmanre@corning.com; Tel.: +1-607-974-2970

**Keywords:** glass, structure, nuclear magnetic resonance, oxide, non-oxide

## Abstract

The study of inorganic glass structure is critically important for basic glass science and especially the commercial development of glasses for a variety of technological uses. One of the best means by which to achieve this understanding is through application of solid-state nuclear magnetic resonance (NMR) spectroscopy, which has a long and interesting history. This technique is element specific, but highly complex, and thus, one of the many inquiries made by non-NMR specialists working in glass science is what type of information and which elements can be studied by this method. This review presents a summary of the different elements that are amenable to the study of glasses by NMR spectroscopy and provides examples of the type of atomic level structural information that can be achieved. It serves to inform the non-specialist working in glass science and technology about some of the benefits and challenges involved in the study of inorganic glass structure using modern, readily-available NMR methods.

## 1. Introduction

Understanding the structure of disordered solids is a challenging endeavor given the lack of long-range periodicity and the inability to make use of standard diffraction methods. However, understanding the short- and intermediate-range structure of glasses is crucial for the development of new compositions with applications in many technological fields. For example, the development of hard, damage-resistant glasses for consumer electronics requires sophisticated balancing between composition and mechanical properties, the former exercising control over the local structure and the latter being largely controlled by this structure. Composition-structure-property understanding in glasses has become critical in nuclear waste sequestration, telecommunications, bioglasses, etc., and represents a large research emphasis in the glass science and technology communities [1,2].

In spite of their inherent structural disorder, glass network structures can be defined on multiple length-scales, and each presents challenges and opportunities for understanding [3]. Short-range structure, basically involving the nearest neighbor bonding and geometry (e.g., cation polyhedra), is most often what is meant by glass structure. This describes the different building blocks of a glass network, and in the case of glasses containing multiple anions such as O and F or O and N, their short-range structure also captures where and how these anions are connected into the network. A longer range structure, on the order of several Angstroms to ~1 nanometer, is described as an intermediate-range structure and essentially describes the interaction or connectivity between short-range structural units. For example, mixing or ordering of silicate and borate polyhedra, or the presence of geometrically well-defined ring or chain structures (e.g., superstructural units), represents the aspects of the intermediate-range structure in glasses. Even a longer-range structure, up to tens of nanometers, can exist in glasses, especially for those exhibiting phase separation or other types of heterogeneities, but such details are even more difficult to characterize, though advances in high energy X-ray diffraction and neutron scattering methods have opened up this important area of structural characterization. While all length-scales of structure in glasses are important, the community has traditionally developed more understanding of short- and intermediate-range structures using a variety of experimental and computational methodologies.

One of the main experimental methods for determining short-range structure in glasses is solid-state nuclear magnetic resonance (NMR) spectroscopy. Based on the pioneering work of Bray and colleagues, NMR experiments on simple borate and borosilicate glasses demonstrated that even in such highly disordered solids, NMR spectroscopy could provide critical insight into their atomic structures. Since these first studies of glass using NMR in 1958 [4], this characterization method has gained many practitioners, substantial advancement in methods and an ever-increasing level of importance in glass science. Today, there are many research groups across the globe using NMR methods to characterize glass structure across all length scales and, more importantly, applying this understanding to develop new glasses or to further understand and optimize many of their important properties. 

A review of all of the many aspects of NMR in glass science would be impossible to accomplish in any single report, so this contribution is focused on a general survey of the many important elements that can be studied using routine NMR spectroscopic methods, arguably the most simple and widespread use of NMR in both academic and industrial studies of glasses. There continue to be a great number of significant and elaborate developments in the technique, including multi-nuclear correlations to expand the length-scale of structural understanding, the powerful combination of modeling and NMR experiments and other areas that lie outside the scope of this work. Past reviews on glass NMR have touched on many of these topics, including the comprehensive examination of NMR for different types of glasses by Eckert in 1992 [5], a recent summary of applications in glass-ceramic materials [6] and various specialized reviews on specific elements of interest or glass families [7,8,9].

As previously mentioned, the first use of NMR to help describe the structure in glasses was at Brown University, where Philip Bray and his students developed measurements and data analysis methods to understand the NMR response of ^11^B and other nuclei in glasses. The initial report in 1958 demonstrated the incredible value of NMR spectroscopy in being able to describe the local bonding environment of cations like boron [4] and eventually led to other elements like silicon, lithium and oxygen. This early work was based on wideline or static NMR and was conducted at fairly low magnetic fields. The resulting data may now seem archaic in terms of resolution or sophistication, but the simple fact that an interpretable signal from a disordered solid could be detected was truly remarkable. Most telling at this time was that the signal could be described using multiple bonding environments for boron, with both three- and four-fold coordination present and controlled by the glass chemistry. In fact, this remains one of the most common applications for NMR in glass science, even after 60 years of technological advances in this field. 

Building on the successful development of glass NMR by Bray, other research groups, most notably that of Müller-Warmuth [10], began to investigate NMR spectroscopy for glass structure determination. The field was significantly advanced in the early 1970s by the development of magic-angle spinning (MAS) NMR of solids, which led to a substantial narrowing of the NMR signal and a concomitant increase in resolution and sensitivity, factors that greatly appealed to researchers investigating glass structure. Further improvements in methodology, especially in commercial instrumentation, magnetic field development, NMR probe technology and sophisticated radio-frequency pulse sequences, continue to this day, driven largely by the increasing application of solid-state NMR in materials science.

## 2. Current State of Glass NMR

**Figure 1:**
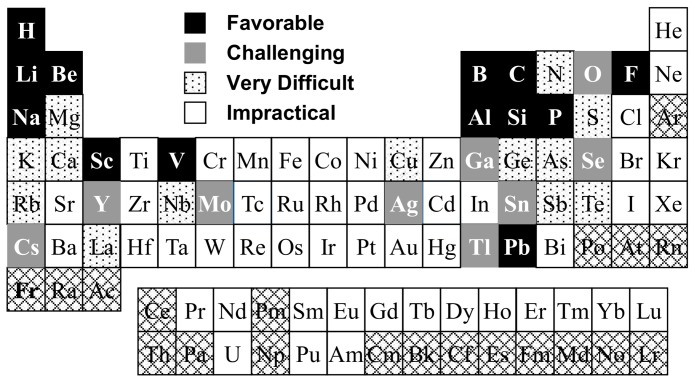
A practical categorization of elements in the NMR study of inorganic glasses. In addition to those identified in the legend, the cross-hatched boxes denote elements that are widely considered impossible for solid-state NMR.

After 60+ years of countless studies and successes, NMR-based understanding of glass structure has advanced to the point where this is an indispensable experimental method for basic glass science and glass technology. Inspired by the efforts of more recent luminaries in the field, including Eckert, Stebbins, Massiot and others, researchers now utilize highly sophisticated solid-state NMR techniques to probe glass structure at different length-scales [8], the structure of glass melts [11], pressure-induced changes to glass structure [12] and with application towards many different chemical elements of interest in inorganic glass formulations [9]. The chart in Figure 1 shows one possible assessment of the elements as studied in glasses using NMR, categorized by ease of study and from personal experience deploying NMR methods in industrial glass research. Those elements highlighted in black boxes are quite common and relatively straightforward to study using widely-accessible solid-state NMR methods (i.e., favorable). Among these are many important network-forming cations, including the aforementioned boron, as well as silicon, phosphorus and aluminum. This “favorable” category also includes a handful of modifier cations (e.g., Li and Na) and some other elements that are common in glasses or easy to characterize. The next category, labeled as “challenging”, is comprised of elements in glasses that have been successfully studied using NMR spectroscopy, but are more difficult to interpret, or require special measurement conditions or protocols. Most notable among these is oxygen, which normally requires enrichment of the glass sample with the ^17^O isotope, making glass samples quite expensive and essentially impossible to implement in large-scale investigations of commercially-relevant glasses. There are then other elements that can be studied with NMR, but are very difficult due to a variety of factors, some of which will be explained later. One example in this “very difficult” category is magnesium, which has an NMR-active isotope (^25^Mg), but when measured in a glass, the signal is unfortunately of poor quality and difficult to interpret. Other elements like germanium (^73^Ge) have very low gyromagnetic ratios, so-called low-gamma nuclei, which presents a variety of challenges, though prospects for NMR of many low-gamma nuclei continue to improve with the development of increasingly large magnetic fields [13]. Finally, there are two general categorizations, which include elements with NMR-active isotopes, but that are essentially impossible to study in disordered solids like inorganic glasses *(*“impractical”), denoted with white boxes, as well as those elements that do not possess an NMR-active spin and are thus completely inaccessible by this spectroscopy (cross-hatched boxes). Based on this general categorization of the “NMR Periodic Table”, these delineations will be used to discuss and review the various applications of NMR spectroscopy towards short-range structural understanding of inorganic glasses. When considering applications of NMR in other areas of materials science, for example research on crystalline solids, the above view of the “NMR Periodic Table” is quite likely different, as the high degree of structural ordering in these materials leads to many benefits and also a few challenges for NMR. There are many published examples of highly successful studies of quadrupolar nuclei in crystalline solids, where the lack of structural disorder leads to higher quality and more easily interpreted NMR spectra. Such aspects of solid-state NMR are outside the intent of this review, but are nonetheless very important. It is also hoped that future research in glasses using NMR spectroscopy will eventually prove much of the assessment in Figure 1 to be incorrect.

### 2.1. Favorable Elements for NMR of Glasses

**Figure 2:**
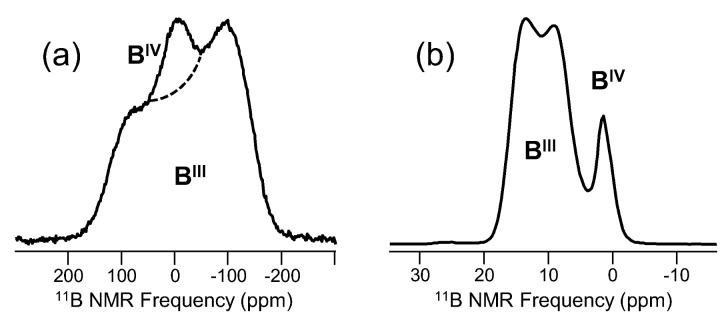
^11^B NMR data for a binary antimony borate glass with composition 40.8Sb_2_O_3_-59.2B_2_O_3_ (mol %) [16], with permission from Society of Glass Technology. The wideline data in (**a**) were obtained at 4.7 T and are displayed in absorption mode, which differs from the original derivative spectra of Bray and other pioneers of glass NMR. The dashed line represents an approximate separation of the two NMR signals. The trace in (**b**) shows the ^11^B MAS NMR spectrum measured at 11.7 T and is typical of most modern high-field NMR data for ^11^B in glasses. In both spectra, the signals from boron in three-fold (B^III^) and four-fold (B^IV^) coordination are labeled. Note the very different frequency scales necessary to plot these data.

Many different elements are important in both ancient and modern glass science, including especially the network-forming cations like silicon. One of these, boron, was the first element studied in glasses using NMR, as described above. Bray and colleagues examined the ^11^B (spin-3/2; 80.42% abundant) and ^10^B (spin-3; 19.58%) NMR signal in a variety of borate and borosilicate glasses, using the substantially different line shapes to describe three- and four-fold coordinated boron polyhedra, and eventually developing the structural models for borate speciation in glasses, which are still important today [14,15]. An example of how standard ^11^B NMR has evolved over the past 60+ years is shown in Figure 2. The static or wideline NMR data used so effectively by Bray and Müller-Warmuth allow for identification of different ^11^B signals, since the quadrupolar coupling constant for four-fold coordinated boron is much smaller than that of the three-fold coordinated sites. In Figure 2a, this is manifested as a relatively sharp resonance superimposed on the broad signal from three-fold coordinated boron. One can readily fit these data to extract the fraction of four-fold coordinated boron (N_4_), which was the basis for much of Bray’s early work on borate and boron-containing glasses. Low field (4.7 T) static NMR of ^11^B in glasses is relatively simple and only requires careful consideration of the nutation behavior of the different ^11^B signals and potential loss or distortion of the broader B^III^ signal due to pulse sequence delay times before acquisition of the free-induction decay (FID). The spectrum in Figure 2a was collected with a 7.5-mm MAS NMR probe (without spinning), using a pulse width of 0.8 μs and recycle delay of 30 s. These data typically require acquisition of hundreds of scans, depending on the boron content of the glass, and thus require only a few hours of acquisition for a suitable signal. Today, MAS NMR has obviated, for the most part, these wideline NMR studies, as improved resolution and simple line shape simulation routines are quite common. The ^11^B NMR spectrum of the same antimony borate glass, but acquired using MAS NMR at an external magnetic field of 11.7 T, is shown in Figure 2b, where almost complete separation of the three- and four-fold coordinated boron resonances is achieved. These data were collected with a 4-mm MAS NMR probe (sample volume = 52 μL), sample spinning of 16 kHz, pulse widths of 0.7 μs and recycle delays of 2 s. A range of 400–1000 acquisitions is sufficient, requiring at most 30 min of experimental time. Data at even higher fields, above approximately 16.4 T, exhibit full separation of these peaks, allowing for easy estimation of the boron coordination in glasses. The impact of different nutation behaviors for B^III^ and B^IV^ sites is especially important when using ^11^B NMR to accurately quantify boron speciation in glasses and is applicable to all types of ^11^B NMR, including both the wideline and MAS data in Figure 2. Practitioners of ^11^B NMR typically utilize very short rf pulses with small tip angles, on the order of π/12 or even as short as π/18. This ensures uniform excitation of all ^11^B spins, regardless of their nutation behavior, and is necessary to collect quantitatively accurate data.

**Figure 3:**
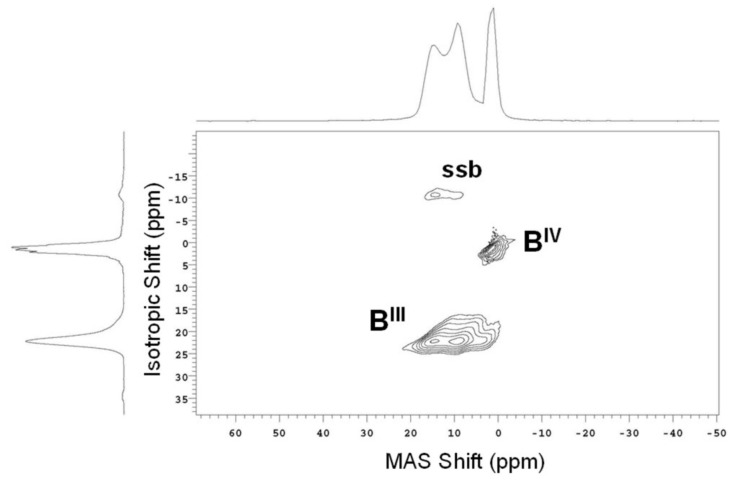
^11^B multiple quantum magic angle spinning (MQMAS) NMR spectrum of a binary sodium borate glass (15.4 mol % Na_2_O), measured at 11.7 T, using a 3.2-mm MAS NMR probe (sample volume ~22 μL). B^III^ and B^IV^ resonances are detected as separate sets of contours, and within the B^III^ signal is evidence for multiple environments corresponding to ring and non-ring B^III^. Traces to the left and top correspond to data projections onto the isotropic and MAS shift axes, respectively. Fine structure in the B^IV^ isotropic projection is from data truncation. Reproduced from [20] with permission from the PCCP Owner Societies.

In the last 20–25 years, other methods have been developed that further narrow the NMR linewidth of quadrupolar nuclei like ^11^B, including double rotation (DOR) [17], dynamic angle spinning (DAS) [18] and multiple quantum magic angle spinning (MQMAS) [19], providing a way to further resolve and characterize different boron polyhedra in glasses. MAS and MQMAS NMR of ^11^B are now commonly used to identity these groups and to accurately follow their compositional, temperature- and pressure-dependent concentrations. An example of ^11^B MQMAS NMR data from an alkali borate glass is given in Figure 3 [20]. These data, which typically take a few hours to collect, provide complete resolution of B^III^ and B^IV^ sites, but also purely isotropic spectra for B^III^ sites, which are broadened by second-order quadrupolar effects under MAS NMR conditions. This allows for better identification of different B^III^ environments, which in boron-rich glasses typically include symmetric B^III^ units in superstructural units (i.e., well-defined rings) and those B^III^ without such geometric constraints (i.e., non-ring boron). ^11^B 3QMAS NMR data can also aid in identifying B^III^ with different numbers of bridging and non-bridging oxygen (NBO), which are often observed in borate glasses with large amounts of modifier. Since boron is indeed a critical element in borosilicate and other commercially-important glasses, ^11^B NMR has remained one of the more common means by which to study glass structure using NMR spectroscopy.

**Figure 4:**
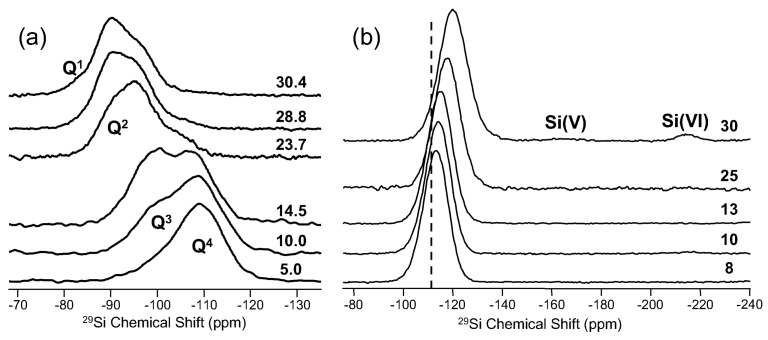
^29^Si MAS NMR spectra for (**a**) a series of binary cesium silicate glasses [25], with Cs_2_O content in mol % to the right of each trace; and (**b**) a series of binary P_2_O_5_-SiO_2_ glasses with mol % P_2_O_5_ shown to the right, adapted from [29] with permission from Cambridge University Press. Q^n^ species are labeled according to their chemical shifts (peak positions) in (**a**), and in addition to the main Q^4^ peak in (**b**), weak NMR signals from five- and six-fold coordinated silicon atoms are marked. The vertical dashed line in (**b**) marks the chemical shift of Q^4^ tetrahedra in pure silica.

Silicon and phosphorus represent two additional glass-formers, which are commonly studied using NMR. ^29^Si (spin-1/2; 4.7%) NMR of glasses was first reported in the early 1970s by Müller-Warmuth [21]. Lippmaa et al. used the newly-invented technique of MAS NMR to characterize the silicate structure in lead silicate glasses [22]. This was followed by other studies of silicate glasses using ^29^Si NMR, including the work of Dupree and co-workers, who estimated the Si–O–Si bond angle distribution from careful analysis of the ^29^Si MAS NMR line shape [23]. More recent advances in methodology have increased the information content from ^29^Si NMR of glasses, and Grandinetti, for example, has used this approach to further describe distributions in short-range structure for silicate groups [24]. An example of using ^29^Si MAS NMR to follow changes in Q^n^ speciation is shown in Figure 4. Here, a series of binary cesium silicate glasses was examined, and there is a clear evolution of ^29^Si chemical shifts with increasing Cs_2_O [25]. In addition to distinct Q^n^ resonances, there are additional peaks in these data, which reflect distinct Q^n^ environments with different next-nearest neighbor (NNN) polyhedra. Careful studies of potassium silicate glasses showed multiple Q^4^ environments at high silica contents, attributed to Q^4^ with only other Q^4^ neighbors and Q^4^ with a mixture of Q^4^ and Q^3^ neighbors [26]. Such structural complexity can be observed experimentally, and more comprehensive descriptions are possible with computational investigations [27]. Static NMR, perhaps considered old fashioned or outdated, can aid in spectral deconvolution of MAS NMR spectra or may be quite useful in the study of complex glasses where the chemical shielding of ^29^Si is further complicated by nearby cations. Although MAS NMR methods are preferred for resolution, the static NMR signal of ^29^Si retains the chemical shift anisotropy (CSA), which is otherwise removed under MAS NMR, and thus, the static line shape can be used to further distinguish between different Q^n^ silicate groups based on symmetry differences. One example of this is for aluminosilicate glasses, where the presence of NNN aluminum has a similar deshielding effect on ^29^Si as does modifier-induced depolymerization via the formation of non-bridging oxygen (NBO). This means, for example, that Q^4^ silicate groups in aluminosilicate glasses having non-zero Al NNN will appear with similar chemical shifts as Q^3^ or even Q^2^ groups surrounded only by other silicate polyhedra [28]. This greatly complicates the interpretation and especially the quantification of different silicate groups and can be aided with better understanding of the CSA exhibited by the different resonances in static ^29^Si NMR spectra. Another example where NNN cations influence the ^29^Si shielding is for phosphorus-containing glasses [29]. In Figure 4b, a series of ^29^Si MAS NMR spectra was measured for binary P_2_O_5_-SiO_2_ glasses. The Q^4^ resonance, which represents all of the silicon polyhedra at low P_2_O_5_, is systematically shifted to a more negative frequency (higher shielding) as these Q^4^ groups are surrounded by a higher average number of phosphate polyhedra. The dashed line marks the position of Q^4^ in pure silica, and it is quite apparent that NNN phosphorus has a marked impact on chemical shift of this peak. At the higher P_2_O_5_ levels, additional silicon species are detected, with small quantities of five- and six-fold coordinated silicon represented by ^29^Si NMR peaks around −160 and −220 ppm, respectively. These higher coordinated silicon groups are charge-balanced by phosphate groups and have been well characterized by both ^29^Si and ^31^P (described in the following) NMR spectroscopies [29]. The Al and P NNN impact on ^29^Si shielding can be even more complicated, especially when both of these network formers are present in the same silicate glass. Although ^29^Si NMR is in principle very simple, interpretation of data in multicomponent glasses is challenging and sometimes impossible. The most notable difficulty in conducting ^29^Si NMR on glassy samples is the lengthy spin-lattice relaxation time (T_1_), which in highly pure silicates can approach tens of thousands of seconds. This means that careful consideration of tip angle and recycle delay are necessary to accurately quantify different silicon species. This typically necessitates a reduced tip angle of π/4 or π/6 and still lengthy recycle delays of hundreds of seconds. The number of acquisitions is then a balance between these requirements and total Si content of the glass, and in many cases, the total experimental time can be numbered in days. One common approach to reduce the ^29^Si T_1_ and total NMR experiment time is the incorporation of a paramagnetic dopant. These are usually a rare earth or transition metal oxide, which can significantly reduce T_1_ in glasses. This approach requires doping of the glass during fabrication and validation that all ^29^Si spins (i.e., sites) are affected by the dopant.

**Figure 5:**
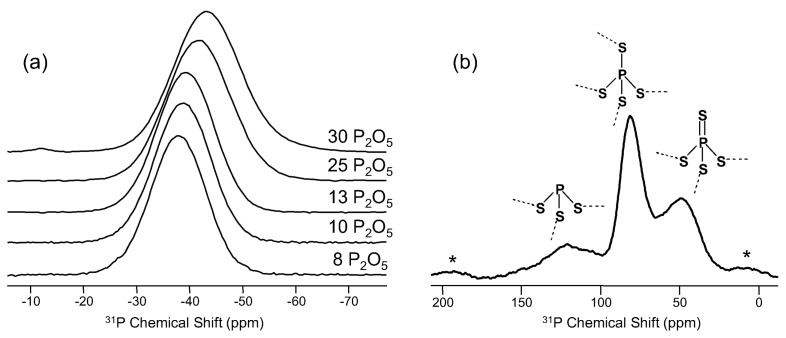
^31^P MAS NMR spectra for (**a**) a series of binary P_2_O_5_-SiO_2_ glasses [29], with permission from Cambridge University Press. P_2_O_5_ content in mol % is shown to the right of each trace. In (**b**) is ^31^P MAS NMR data for a ternary sulfide glass with composition 10P_2_S_5_-6.7Ga_2_S_3_-83.3GeS_2_, adapted from [37] with permission from Elsevier. Different P polyhedra and their associated ^31^P NMR resonances are labeled in (**b**), and asterisks denote spinning sidebands.

As with silicon, phosphorus has been well studied using NMR and tends to exhibit similar trends with composition. ^31^P (spin-1/2; 100%) NMR of glasses was first demonstrated in the mid-1980s by Kirkpatrick and co-workers, using this technique to examine the phosphorus speciation in silicate glasses [30]. There are currently several groups who specialize in ^31^P NMR of glasses and who use their expertise to develop multi-nuclear correlations with other elements or to use more advanced methods to probe phosphate structure at longer length-scales than the simple phosphate groupings of Q^n^ polyhedra [31,32]. The chemical shielding of ^31^P is sensitive to phosphate group polymerization (i.e., number of NBO), so accurate study of simple phosphate glasses is well established. The NMR response of ^31^P in more complex glasses is also influenced by neighboring polyhedra like Al or Si, so resolution of different Q^n^ phosphate groups is substantially reduced in these cases [33]. The shielding of phosphate groups has also been shown to be very sensitive to the nature of the charge-compensating cations [34], and through detailed studies of phosphate crystals, this information can be leveraged to better understand the phosphate and modifier environments in complex phosphate glasses, including especially mixed modifier bioglasses [35]. The data in Figure 5a represent ^31^P MAS NMR spectra for a series of binary P_2_O_5_-SiO_2_ glasses, exhibiting changes in chemical shielding of the ^31^P nucleus with glass composition. In these glasses, phosphorus is primarily found in Q^3^ tetrahedra, and the change in chemical shift in Figure 5a is reflective of mixing between Q^4^ silicate and these Q^3^ phosphate groups. The impact of intermediate-range ordering on both ^31^P and ^29^Si (cf. Figure 4b) shielding is such that these types of correlations can be used to examine phase separation in P_2_O_5_-SiO_2_ glass prone to devitrification [29]. ^31^P NMR has not been limited to oxide glasses, but is also a commonly-studied element in non-oxide and glasses containing more than one anion. Eckert has extensively studied phosphorus structure in various chalcogenide glasses and, in many cases, used the CSA information to make spectral assignments [36]. The data in Figure 5b demonstrate some of the spectral features associated with phosphorus in sulfide glasses, where peaks belonging to three different coordination environments are clearly seen [37]. These phosphorus sulfide groups are sensitive to glass composition and exhibit concentrations that depend on the amount of Ga_2_S_3_, which associates with PS_4_ tetrahedra to form symmetric GaPS_4_-type environments, similar to the AlPO_4_- and BPO_4_-like associated polyhedra in oxide glasses [38,39]. Phosphorus speciation in oxyfluorides or oxynitride glasses is also known from NMR study and, for the latter materials, used to ascertain the extent of bonding between phosphorus and nitrogen [40]. Overall, ^31^P NMR in glass science is relatively simple. Natural abundance is high, so glasses containing even small quantities of P (thousands of ppm) can be studied. As with ^29^Si, the T_1_ of ^31^P spins in glasses can be quite long, though not nearly as lengthy as ^29^Si. Therefore, the combination of short tip angles and recycle delays on the order of 60–300 s are usually sufficient to collect these data. The experiments in Figure 5a were made at 11.7 T with a 5-mm MAS NMR probe (sample volume ~160 μL) and, using π/4 pulse widths and 15-s recycle delays, were usually collected in 4–8 h. The data in Figure 5b required additional considerations. These ^31^P NMR experiments were made at 4.7 T, using a 5-mm MAS NMR probe and sample spinning of 10–12 kHz. As seen in Figure 5b, there are spinning sidebands near the isotropic resonances, and a larger sample size and concomitant slower spinning would mean that these sidebands would overlap the peaks of interest. Similarly, making such measurements at a higher magnetic field would require faster sample spinning and thus smaller samples to avoid this complication. Recycle delays in these chalcogenide glasses are longer (~300 s), even when using π/4 tip angles, so measurements were typically made over the course of 12–24 h. These two ^31^P NMR examples serve to demonstrate that different glass compositions and different MAS NMR experiments require careful determination of these experimental parameters necessary to acquire high quality data. These examples also demonstrate the potential benefit of conducting some NMR experiments at lower magnetic field strengths; another aspect of solid-state NMR that appears to be out of favor.

**Figure 6:**
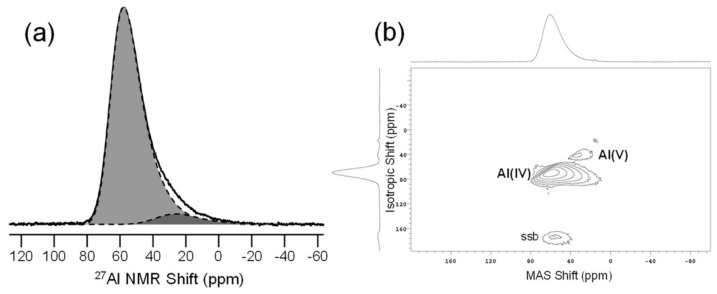
^27^Al NMR data for a glass with composition 24CaO-26.3Al_2_O_3_-49.7SiO_2_ [44]. (**a**) ^27^Al MAS NMR spectrum measured at 16.4 T, with data in bold line and fitting of Al(IV) and Al(V) polyhedra denoted by dashed lines and filled curves; (**b**) ^27^Al 3QMAS NMR spectrum obtained at 16.4 T, with Al(IV) and Al(V) peaks marked. A very small signal from Al(VI) in the zirconia rotor (background) is also seen in the upper right of the contours. Plots to the top and left represent projections onto the MAS and isotropic shift axes, respectively.

Aluminum, while considered a network former in glasses, can exhibit much more complexity than the traditional glass formers above. We know from a variety of studies that aluminum is charge-balanced in four-fold coordination by glass modifiers (e.g., alkali or alkaline earth cations), but if these are insufficient, then aluminum can take on higher coordination numbers to satisfy charge-balancing [41]. Thus, ^27^Al (spin-5/2; 100%) NMR has developed into one of the most important uses of NMR in glass science. First described by Kirkpatrick and colleagues in the 1980s, the application of moderately high magnetic fields and MAS NMR provided the resolution of different aluminum resonances [42]. These early studies were further complicated by the presence of what is now accepted as Al in five-fold coordination, a rather unusual environment in Al-bearing minerals. Resolution of these polyhedra, and especially accurate quantification of their abundances, has continued to improve with the development of higher magnetic fields and techniques like MQMAS NMR. Now, it is quite common to study ^27^Al using MQMAS and MAS NMR at high magnetic field strengths, greater than 14 T, yielding highly resolved spectra and enabling additional understanding of Al in glasses [43]. As an example, consider the ^27^Al NMR data in Figure 6, obtained for a calcium aluminosilicate glass with the analyzed composition of 24CaO-26.3Al_2_O_3_-49.7SiO_2_, measured at 16.4 T using a 1.6-mm MAS NMR probe with sample spinning of 25 kHz and a sample volume of 12 μL [44]. With π/12 tip angles (0.6 μs pulse width) and 2-s recycle delay, ^27^Al MAS NMR spectra such as in Figure 6a can be acquired in 30–60 min. The ^27^Al 3QMAS NMR experiment in (b) was made using the same probe configuration and typically requires 4–8 h of instrument time. The ^27^Al MAS NMR spectrum in Figure 6a shows a broad, asymmetric resonance from AlO_4_^−^ tetrahedra, which are charge-balanced by Ca^2+^. Even in a glass, which is nearly completely charge-balanced (i.e., CaO = Al_2_O_3_), there are clear indications for Al in higher coordination. Accurate line shape simulations, as used in Figure 6a, allow some quantitative assessment of the Al speciation, indicating that the Al(V) population of 6.3 atom % is greater than expected for this slightly peraluminous glass composition. One can also utilize ^27^Al 3QMAS NMR spectroscopy to qualitatively identify the different Al sites in these oxide glasses. Here, in Figure 6b, there is clear evidence for a weaker set of contours, assigned to the Al(V) polyhedra that were also detected in the MAS NMR data. The combination of MAS and 3QMAS NMR data is invaluable in describing the short-range structure of such glasses.

**Figure 7:**
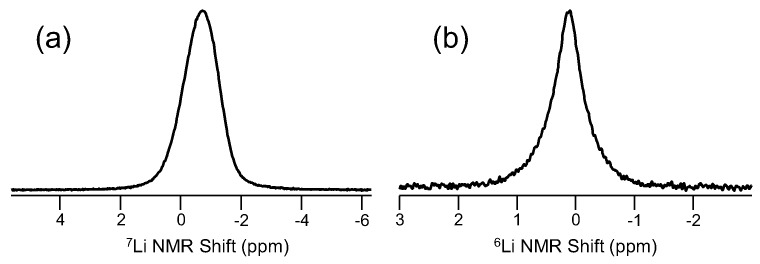
Representative (**a**) ^7^Li and (**b**) ^6^Li MAS NMR spectra for lithium aluminosilicate glasses [49]. Note the different shift scales used for these data.

Other commonly-studied elements in Figure 1 having application in glass science include lithium and sodium, two important modifiers in glass, as well as fluorine, which next to oxygen is one of the most commonly-found anions in inorganic glasses. Lithium NMR, using ^7^Li (spin-3/2; 92.58%), was demonstrated in glass science again by Bray, showing in the early 1970s the ability to study ionic motion in glasses [45]. Müller-Warmuth also demonstrated early the importance of ^7^Li NMR in glasses [46]. More recently, ^7^Li and ^6^Li (spin-1; 7.42%), both of which exhibit favorable NMR properties, have been used to address a variety of topics, including ionic motion, short-range structure and elemental partitioning in glass-ceramic systems. ^7^Li generally exhibits a broader signal due to larger quadrupole moment, but is more abundant and has a higher resonance frequency. ^6^Li is essentially like a spin-1/2 nucleus as a result of a very small nuclear quadrupole moment and has been shown to give higher resolution (e.g., narrower peaks) and thus sometimes used to examine lithium environments in glasses [47]. One example for both ^6^Li and ^7^Li MAS NMR for a lithium aluminosilicate glass, as drawn in Figure 7, exemplifies the type of NMR spectra achievable for these modifier cations. The ^7^Li MAS NMR spectrum contains a single resonance, which is slightly asymmetric as a result of some residual second-order quadrupolar broadening. The ^6^Li MAS NMR data (Figure 7b) are typically more symmetric and narrower, which in principle allows for enhanced spectral resolution and the ability to discriminate between different Li^+^ sites, though the experimental trade-offs are reduced sensitivity, due to lower natural abundance, and much longer T_1_ relaxation. For example, these Li NMR spectra were collected at 16.4 T using a 3.2-mm MAS NMR probe, but the ^6^Li MAS NMR spectrum required short pulse widths (π/6) and 600-s recycle delays to allow for relaxation, while ^7^Li MAS NMR data were collected much faster (π/6, but only 10-s recycle delay). On top of this drastic difference in spin relaxation, the natural abundance of the two Li isotopes makes ^6^Li MAS NMR even more time consuming, usually requiring hours to collect an adequate signal. Another feature of the spectrum in Figure 7b is the Lorentzian line shape, reflecting Li ion mobility in this room temperature measurement, which is also why ^6^Li and ^7^Li NMR have found significant use in the study of Li-conducting glasses [48]. In addition, ^6^Li NMR studies have been particularly useful in following lithium partitioning in glass-ceramics [49].

**Figure 8:**
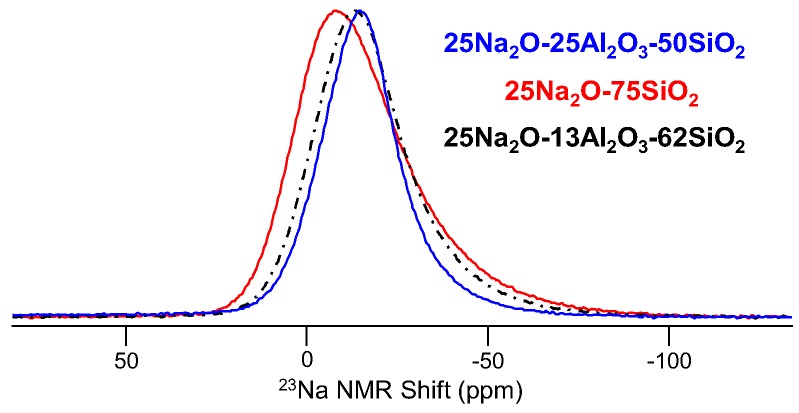
^23^Na MAS NMR spectra measured at 11.7 T for three different silicate glasses.

Sodium (^23^Na (spin-3/2; 100%)) is interesting in that the signal is easy to measure, the quadrupolar coupling is modest and NMR of ^23^Na can further benefit from high-resolution techniques like MQMAS. However, in glasses, sodium has a rather poorly-defined coordination environment. We generally consider sodium to have an average coordination number between five and six, or even higher in glass melts, which has been well studied using ^23^Na NMR [50,51], but the ^23^Na NMR spectrum of a glass reflects only the average environment, which while very important in glass science, does not yield the resolution of different structural or chemical environments. The ^23^Na MAS NMR spectra in Figure 8 show how line shape and frequency change with glass composition, reflecting the role of Na^+^ as either a modifier (i.e., forms NBO) or a charge-balancer for negatively-charged AlO_4_^−^ polyhedra; however, even in a glass containing both sodium environments, for example the peralkaline 25Na_2_O-13Al_2_O_3_-62SiO_2_ glass in Figure 8, only one peak is detected, and it appears intermediate to the peaks due to Na^+^ in either a charge-balancing or network modifier role. MQMAS NMR of these same glasses does not yield improved resolution in the sense of identifying multiple peaks and thus multiple sodium sites, but are still invaluable in characterizing isotropic chemical shift and quadrupolar coupling constants for the average sodium environment, both of which can be correlated with coordination number and average sodium-oxygen distance [52]. The latter has been exploited in studying the impact of pressure on the structure of sodium-modified glasses [12,53]. ^23^Na MAS NMR data like those in Figure 8 are collected using π/12 pulse widths, 1- or 2-s recycle delays and the acquisition of several hundred scans, depending on the Na content of the glass. These particular data were measured at 11.7 T with a 3.2-mm MAS NMR probe (20-kHz sample spinning), and each spectrum required approximately 30 min of experimental time.

**Figure 9:**
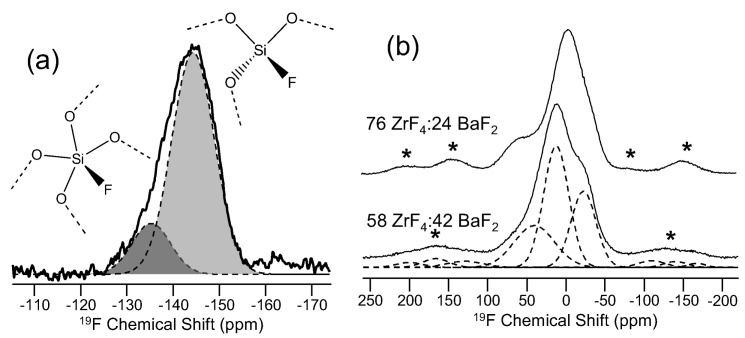
^19^F NMR data for (**a**) F-doped silica [56], with permission from Elsevier, and (**b**) two binary barium fluorozirconate glasses, adapted with permission of Elsevier from [58]. In (**a**), the assignment of the two resonances is depicted in the structure diagrams, where all oxygen atoms are bridging to other silicon atoms (not shown in diagram). Glass compositions and spinning sidebands (asterisks) are shown for (**b**). In both figures, solid lines denote experimental ^19^F MAS NMR data, and filled curves or dashed lines represent fitting of the data using Gaussian lineshapes.

^19^F (spin-1/2; 100%) NMR has been used to study the bonding of fluorine to different cations in complex glasses, generally showing a preference for aluminum [54]. Bray (again) was the first to demonstrate ^19^F NMR of glass [55], and advances in MAS NMR have given us the ability to detect and study small quantities of fluorine in doped glasses such as silica for optical telecommunication [56], oxyfluoride glass-ceramics [57] and also in fluoride glasses [58]. The latter are examples of oxygen-free glasses, where fluorine environments can be quite complex though well understood using ^19^F NMR spectroscopy. The data in Figure 9 represent ^19^F MAS NMR spectra for either an oxyfluoride glass, such as F-doped silica, or from a series of fluoride glasses. The data in Figure 9a were used to identify two fluorine bonding configurations upon addition of fluorine to SiO_2_ glass. The two fluorine resonances, assigned to single Si-F bonds on four- and five-fold coordinated silicon groups, were further characterized with ^29^Si NMR and ^19^F→^29^Si cross-polarization MAS (CPMAS) NMR (data not shown) [56]. In the study of fluorozirconate glasses, comparison with ^19^F NMR data from crystalline fluorozirconates and careful evaluation of the ^19^F CSA for the different sites provide identification and quantification of three distinct fluorine environments in these glasses [58]. In both examples, ^19^F NMR required significant measurement time, due either to low F-content of the F-doped silica glass in (a) or, for both studies, lengthy ^19^F T_1_ values. Both sets of experiments were conducted at 4.7 T using a 4-mm MAS NMR probe (17 kHz sample spinning) for the data in (a) and a 2.5-mm MAS NMR probe (sample spinning of 30 kHz) for (b). This choice of probes was dictated by the MAS NMR spinning rates necessary to fully separate spinning sidebands from isotropic peaks, which is often the case for solid-state NMR of glasses and other solids, but balanced by the need to maximize sample volume and, hence, sensitivity. The data in (a) involved π/4 pulse widths and recycle delays of 1800–3000 s, corresponding to many days of signal acquisition. The studies of fluorozirconate glasses, enabled by the high F content, allowed for much less signal averaging, and recycle delays of 60–90 s were suitable for full relaxation of the spins. 

The other “common” or favorable elements in Figure 1 are not necessarily common in their use or importance in glass science, but rather are easily studied elements using standard NMR methods. Two such examples would be hydrogen (^1^H (spin-1/2; 99.98%)) and carbon (^13^C (spin-1/2; 1.108%)), both of which are ubiquitous in organic chemistry, but rarely studied in an inorganic glass. However, both of these are critical to an area of research that has been active for many decades: the surface modification of inorganic oxides. Glasses, in particular, have been coated with silane and other chemistries, for which the covalent attachment to surface sites has been studied using a variety of NMR techniques. Most common is CPMAS NMR, which uses the abundant signal from ^1^H to detect ^13^C or ^29^Si spins in the attached molecule or glass surface. This method, widely utilized by Maciel and others [59], allows for the study and optimization of glass surface chemistries for a variety of applications. More recently, similar approaches have been used to understand glass corrosion by aqueous media, examining the proton interaction with other elements like ^29^Si, ^31^P and ^23^Na, all to better understand glass corrosion [60] and nuclear waste sequestration [61], to name just a couple of prominent uses. There are also some key examples of where ^1^H NMR has been used to study the hydrogen environment in bulk inorganic glasses. There are several pioneering studies of water in glass using ^1^H NMR [62,63,64,65]. Many glasses are inherently wet due to methods of fabrication or poor chemical durability, so ^1^H NMR has provided information on hydroxyls, including concentration and bonding environment (i.e., SiOH vs. AlOH, etc.). Some glasses are also intentionally loaded with H_2_ gas for optical performance, and the reaction of hydrogen with defects in the glass structure can be evaluated using ^1^H NMR [66]. Early studies were hampered by strong dipolar coupling between protons, which could not be removed with modest MAS NMR sample spinning. Recent advances in MAS NMR probe design, with spinning rates exceeding 100 kHz, may alleviate this source of line broadening and provide even more information on hydrogen in glassy systems, though to achieve such high sample spinning rates, rotor diameters and samples volumes are becoming increasingly smaller.

Vanadium (^51^V (spin-7/2; 99.76%)) and lead (^207^Pb (spin-1/2; 22.6%)) have also been studied in glasses using NMR. ^51^V NMR is fairly simple in terms of NMR receptivity (e.g., natural abundance and resonance frequency), but is complicated by both quadrupolar coupling and large CSA. Application of ^51^V NMR in glass science was first demonstrated in 1970 [67], and more recent studies have extensively characterized vanadium in glasses, leading to an enhanced understanding of the NMR response and its relation to the glass structure [68]. NMR of lead in glasses, studied many years ago by Bray [69], has also seen limited application in this field. The signal is readily detected, but complicated and made further difficult by the different roles of lead in glasses. In some systems, lead is a classic modifier, for example in lead borate glasses, but other compositions contain lead that also acts as a network former [70]. ^207^Pb NMR can be used to study these different characteristics of lead in glass, but its importance seems to have diminished over the years.

**Figure 10:**
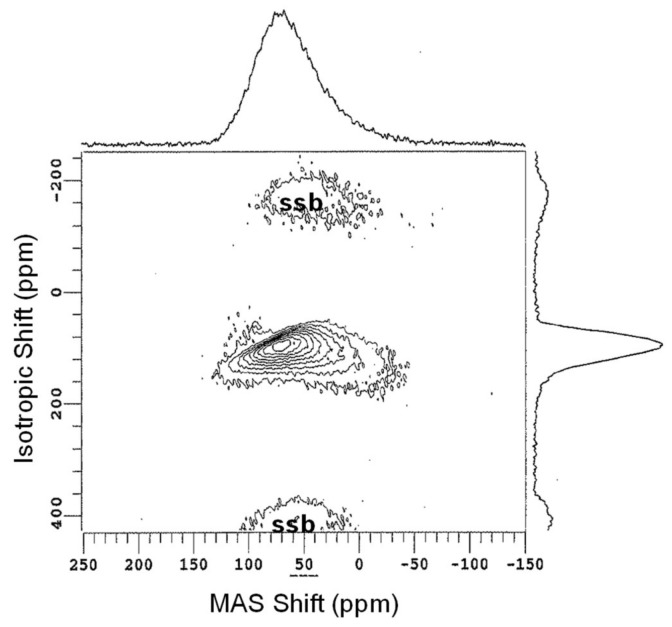
An example of ^45^Sc 3QMAS NMR (at 11.7 T) data for a Sc-containing sodium silicate glass (15Sc_2_O_3_) [71]. Isotropic and MAS projections are shown to the right and top, respectively. Average position of the ^45^Sc resonance in these two dimensions can be used to determine isotropic chemical shift and quadrupolar coupling product, the latter related to the ^45^Sc quadrupolar coupling constant [76]. Data were collected using a 3.2-mm MAS NMR probe, with a sample volume of 22 mL and spinning of 20 kHz. Both ^45^Sc MAS (data not shown) and 3QMAS NMR data were acquired relatively quickly, with adequate data collected in several hours for each sample.

Finally, in terms of easily measured elements in glass, scandium (^45^Sc (spin-7/2; 100%)) and beryllium (^9^Be (spin-3/2; 100%)) are two that have not been exploited to the same level as even some of the less studied elements above. ^45^Sc NMR is very easy using widely-accessible magnetic fields and probes, but has less utility in glass science, especially in terms of commercial glasses. In spite of this limited interest, several groups have recently reported ^45^Sc NMR studies of glasses [71,72,73], and as shown in Figure 10, even ^45^Sc MQMAS data can be readily measured for glasses. Here, for a Sc-containing sodium silicate glass, the MAS and 3QMAS NMR data provide information on ^45^Sc chemical shielding and also quadrupolar coupling constants. These parameters can then be correlated with the short-range structure in the glass, including approximate coordination numbers for the scandium cation. ^9^Be is also easy to study using standard NMR techniques and forms excellent oxide and fluoride glasses, but due to concerns about toxicity, has also been rather limited. ^9^Be NMR data from glasses were initially reported by Bray [74], and there are several more recent NMR studies of beryllium borate glasses [75] and amorphous BeF_2_, which is isostructural with pure silica [74].

### 2.2. Challenging Nuclei in Glass

Next in terms of experimental ease and adoption in glass science are elements with favorable NMR properties, some of which are even quite common in other scientific fields, but nonetheless are challenging due to a variety of factors, including the fact that low symmetry and site distributions in disordered solids lead to poorly-resolved or difficult to detect NMR signals. 

**Figure 11:**
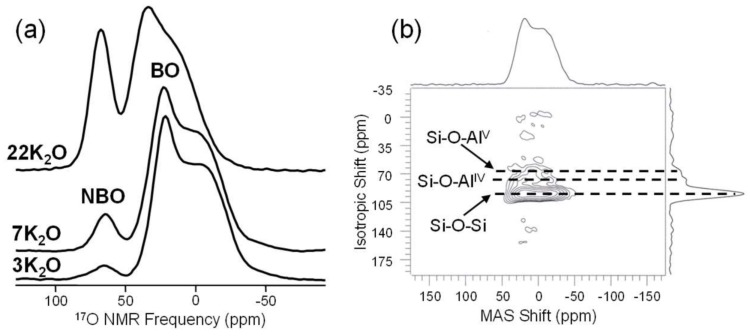
Examples of ^17^O NMR in glass science. The series of MAS NMR spectra in (**a**) show the evolution of non-bridging oxygen (NBO) concentration as a function of K_2_O in binary potassium silicate glasses, adapted with permission by Elsevier from [26]. The ^17^O 3QMAS NMR spectrum of (**b**), measured for a 10 wt % Al_2_O_3_ in silica glass, provides the resolution of multiple bridging oxygen sites, which differ due to the nature of their connected network-forming polyhedra, as shown by the dashed lines and labels. Adapted with permission from [80]. Copyright 2004, American Chemical Society.

Oxygen, somewhat anomalous for this category, is quite popular and relatively easy to interpret, but due to the very low natural abundance of ^17^O (spin-5/2; 0.037%), must usually be enriched in the sample of interest. This has been done superbly by groups like that of Stebbins, who have used ^17^O NMR to study connectivity between cations, glasses at elevated temperatures and other important topics in glass science [77,78]. Once enriched with the ^17^O isotope, both standard and advanced techniques, including MQMAS, can be used to detect and resolve different oxygen sites in glasses. Sophisticated studies of ^17^O parameters related to structural aspects, such as isotropic chemical shift and quadrupolar coupling constants, have been conducted by Grandinetti to better understand the NMR response and especially the distribution of oxygen bond angles in oxide glasses [79]. Simple studies of bridging and non-bridging oxygen populations are quite useful, especially in multicomponent glasses where structural models are either incomplete or inaccurate. For example, the detailed study of short- and intermediate-range structure in binary alkali silicate glasses can be furthered using ^17^O MAS NMR to accurately quantify the NBO content, as demonstrated in Figure 11a. These spectra, measured on binary potassium silicate glasses, clearly exhibit resolution between bridging and non-bridging oxygen sites, and since these experiments are highly quantitative, the peak areas are used to determine NBO concentrations in glasses [26]. Data such as these can be measured at modest field strength (11.7 T for Figure 11a) and, depending on the ^17^O enrichment, take little experimental time to collect. The data in Figure 11a were acquired using 0.6-μs excitation pulses (<π/4), 1-s recycle delays and signal averaging of 8000–58,000 scans. Other glasses, especially those with multiple network formers or a mixture of modifier cations, exhibit much more complex ^17^O NMR data, as different BO and NBO environments substantially overlap in ^17^O MAS NMR spectra, but due to different shielding and/or quadrupolar coupling constants, can be better resolved in the 3QMAS NMR experiment [80,81]. This is demonstrated for binary Al_2_O_3_-SiO_2_ glasses in Figure 11b, where the ^17^O 3QMAS NMR data contain partially-resolved peaks from bridging oxygen between different network-forming polyhedra. Such studies have significantly advanced our understanding of different oxygen environments in glasses, including even the study of tricluster oxygen, bound to three different network-forming polyhedra [82]. These types of two-dimensional experiments naturally mean longer experiments, and as is the case for ^17^O MAS NMR, the total experimental time depends largely on the isotope enrichment. For the data in Figure 11b, enrichment was based on using 80% ^17^O-enriched water to make labeled SiO_2_, which was subsequently utilized in melting the binary aluminosilicate glass. This level of labeling, as well as the incorporation of a small amount of Gd_2_O_3_ to facilitate spin relaxation, enabled the data in Figure 11b to be collected in 70 h (6000 scans were collected for each of 60 t_1_ values, using a recycle delay of 0.7 s). Sacrificing signal/noise, working at a higher magnetic field or achieving higher ^17^O-enrichment could each lead to shorter total experiment times.

**Figure 12:**
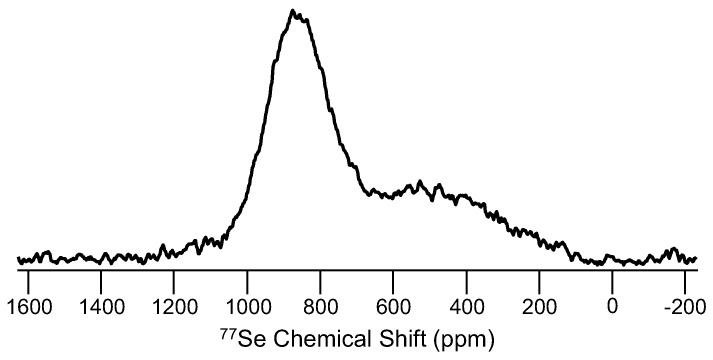
^77^Se MAS NMR spectrum of a binary germanium selenide glass (Ge_10_Se_90_), with two distinct resonances assigned to Se bridging two Se atoms (left) and Se bridging one Se and one Ge atom (right) [85]. This room temperature spectrum was obtained at 4.7 T, using a 4-mm MAS NMR probe with modest sample spinning (10 kHz) and a recycle delay of 60 s, accounting for the lengthy ^77^Se spin-lattice relaxation time.

**Figure 13:**
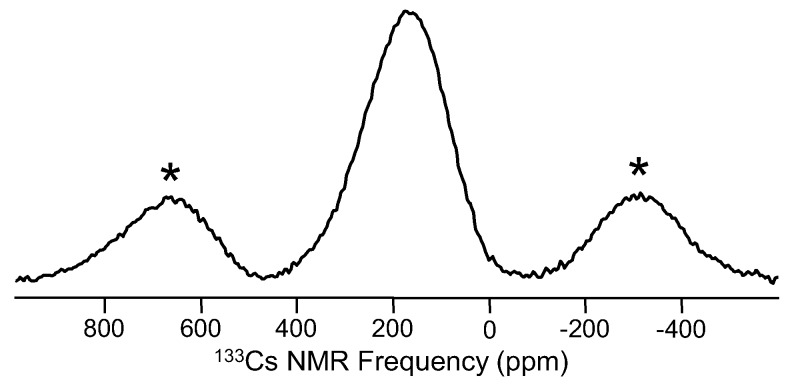
^133^Cs MAS NMR spectrum of a glass having a composition of 25Cs_2_O-37.5Al_2_O_3_-37.5Ga_2_O_3_ [87]. Spinning sidebands are marked by asterisks. Data were collected at 11.7 T using a 2.5-mm MAS NMR probe (sample volume = 11 μL; spinning rate = 30 kHz), with a total acquisition time of 1–2 h.

**Figure 14:**
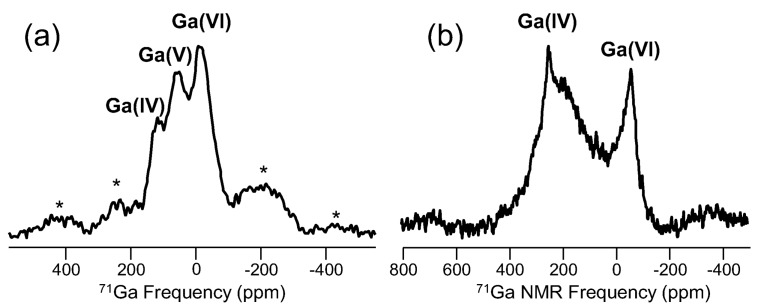
^71^Ga MAS NMR spectra for (**a**) a ternary sodium gallium tellurite glass (10Ga:10Na:80Te in atom %) and (**b**) a sulfide glass with composition 90AsPS_4_-10GaPS_4_ [89]. Labels represent different gallium coordination numbers, and spinning sidebands are marked by asterisks. Both spectra were collected at 11.7 T using a 2.5-mm MAS NMR probe with sample spinning of 30 kHz. π/12 pulse widths, 0.5-s recycle delays and number of acquisitions (as high as 400,000) scaled by the Ga_2_O_3_ content of the glass resulted in total experiment times of ~12–60 h for each glass sample.

Selenium, gallium and cesium NMR studies in glasses are somewhat common, though each of these elements presents its own challenges. ^77^Se (spin-1/2; 7.58%), widely used in the structural characterization of Se-containing chalcogenide glasses, exhibits broad peaks and suffers from lengthy spin-lattice relaxation times. Natural abundance of ^77^Se is decent, and in selenide glasses, the concentration of ^77^Se is high, so MAS NMR studies have been quite useful in understanding element mixing in binary and ternary selenides, as well as network dynamics in glasses characterized with low dimensionality, including pure amorphous selenium. The first published report of ^77^Se NMR of a glass was in the early 1970s and was focused on glassy Se and As_2_Se_3_ [83], two systems that continue to be studied using NMR. Much like the case of oxygen described above, the main application of ^77^Se NMR is to characterize the different bonding configurations between network polyhedra, especially homopolar and heteropolar bonding between various metal cations and Se, as demonstrated for binary Ge_x_Se_100−x_ glasses like that in Figure 12. Models for arsenic selenides, germanium selenides and other glasses have benefitted from accurate, quantitative assessment of selenium environments [84]. Interestingly, many of these selenide glasses have fairly low T_g_ values, so variable temperature ^77^Se NMR has been used to follow network dynamics, correlating different T-dependent line shapes with structural and viscous relaxation [85]. Cesium NMR (^133^Cs (spin-7/2; 100%)), which represents another alkali oxide modifier, has been used especially to understand the cesium environments and crystallization in glasses for nuclear waste containment [86]. ^133^Cs NMR peaks from glasses are broad and give rise to significant spinning sideband intensities, as can be seen in the ^133^Cs MAS NMR data of Figure 13. In addition, the change in frequency for these peaks as a function of glass composition is small [87], and thus, this particular element has not been widely studied in glasses using NMR. Gallium, which also has some challenges due to a moderate quadrupole moment, is thought to exhibit structural chemistry similar to aluminum and thus has been examined using ^71^Ga (spin-3/2; 39.6%) NMR. ^71^Ga MAS NMR experiments confirm four-fold coordination in many different glasses, similar to how modifiers charge-balance AlO_4_^−^ groups. In some glasses, higher coordinated gallium sites have been identified, but are difficult to fully resolve from the prevalent Ga(IV) peak. Unlike ^27^Al and a few other quadrupolar nuclei, one cannot utilize MQMAS NMR techniques to improve the spectral resolution for ^71^Ga. To our knowledge, due mostly to the magnitude of the quadrupolar coupling constant, there are no reported successes in measuring an MQMAS NMR spectrum of ^71^Ga in a glass. Still though, interest in gallium NMR of glasses continues, and with increasing magnetic fields being developed, advances will continue to be made. Figure 14 shows spectra for some different Ga-containing glasses, where sufficient resolution from ^71^Ga MAS NMR at 11.7 T allows for identification of different gallium coordination numbers. In Figure 14a, Ga(IV), Ga(V) and Ga(VI) peaks are identified, and their relative concentrations are quite similar to aluminum in the analogous Al-containing tellurite glasses [88]. The example in Figure 14b, obtained from a Ga-containing chalcogenide glass, is one of the few examples where six-fold coordinated Ga has been observed in a non-oxide glass [89].

**Figure 15:**
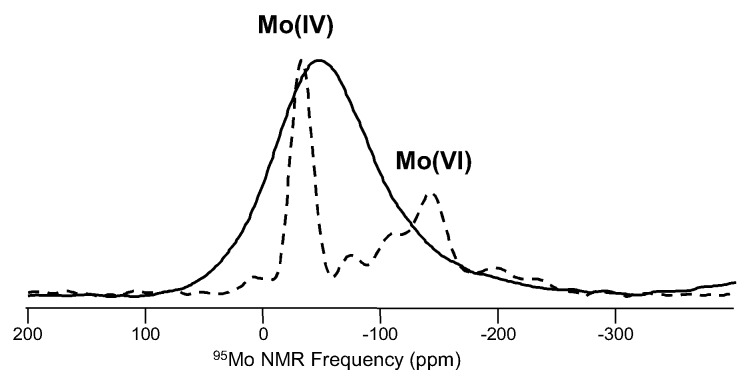
An example of ^95^Mo MAS NMR from a mixed-alkali molybdate glass (33.3R_2_O·66.7MoO_3_), compared with data from polycrystalline Na_2_Mo_2_O_7_ (dashed line) [93]. Comparisons with NMR data from known crystalline compounds are beneficial for understanding data from glasses. ^95^Mo NMR peaks from tetrahedral and octahedral Mo are shown for Na_2_Mo_2_O_7_, and baseline “ripples” for these data are from truncation of the time-domain signal from incomplete acquisition of the full spin echo.

Other uncommon or challenging NMR nuclei from Figure 1 include silver and molybdenum. The former has actually been extensively studied in Ag^+^ conducting glasses, where ionic mobilities are fairly high. The earliest mention of ^109^Ag (spin-1/2; 48.18%) NMR in published glass science studies, appearing in 1986 from several different groups, involved such applications [90,91]. Otherwise, interest in deploying NMR to study silver in glasses has been limited. Molybdenum NMR (^95^Mo (spin-5/2; 15.72%)) of glasses is relatively recent in the literature, as this low-gamma nucleus has benefitted from the availability of larger magnetic fields. Molybdenum is not a common element in commercial glasses, so there are very few examples thus far, and these have been connected to Mo solubility concerns for nuclear waste storage [92]. Other studies, as shown in Figure 15, have examined Mo speciation in simple alkali molybdate glasses, where comparison with known crystalline compounds like NaMoO_4_ and Na_2_MoO_7_ have shown the presence of Mo in both four-fold and six-fold coordination, and the average coordination number changes with glass composition [93]. These NMR data require specialized NMR probes, or tuning configurations, to reach the low Larmor frequency of ^95^Mo, which is 32.6 MHz at a field strength of 11.7 T. Data were collected at sample spinning rates of nominally 12 kHz in a 5-mm MAS NMR probe. In order to avoid capacitor ringing issues that accompany NMR work at low Larmor frequencies, ^95^Mo NMR data were collected using whole-echo acquisition, with a delay time of 1 ms between pulses and a recycle delay of 10 s. ^95^Mo MAS NMR data were typically collected with averaging of 8000–20,000 scans, corresponding to total experiment times up to approximately 60 h. 

**Figure 16:**
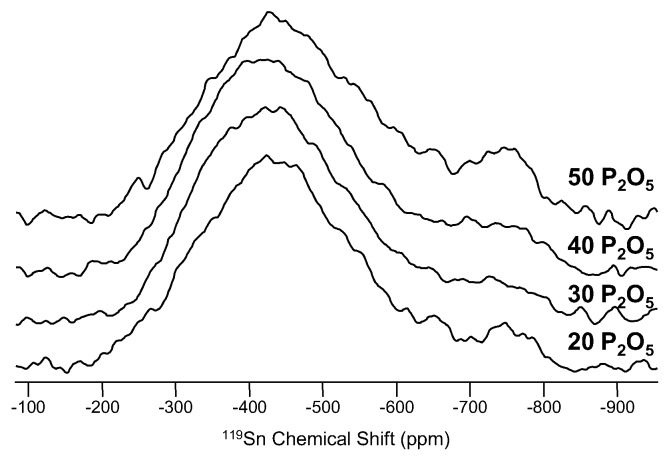
Wideline ^119^Sn NMR spectra, measured at 11.7 T, for a series of tin fluorophosphate glasses ((60 − x)SnF_2_-40SnO-xP_2_O_5_) with P_2_O_5_ contents given to the right of each spectrum. The main feature around −450 ppm is from Sn^2+^ and is relatively unaffected by glass composition. A weak resonance around −750 ppm, assigned to Sn^4+^, is detected in several glasses and is most intense at the highest P_2_O_5_ content [96].

The other elements in this category, tin, thallium and yttrium, have been shown to be of marginal interest in NMR studies of glasses. Thallium (^205^Tl (spin-1/2; 70.5%)) has very high sensitivity, and NMR of this element in glass was demonstrated first by Bray in 1977 [94]. Thallium has only limited interest in glass science and, as of late, is not something we generally consider to be an important component; therefore, NMR studies of thallium are very limited. Tin (^119^Sn (spin-1/2; 8.58%)) is ubiquitous in commercial glasses, either used as a fining agent to eliminate gas bubbles or as a molten support for the float process of glass manufacturing. This provides many opportunities to study tin in glass, but these particular examples involve only minor quantities of tin, making Sn NMR quite difficult. Very few studies of Sn in glass using NMR techniques appear in the scientific literature, but one such investigation of tin-containing silicate glasses was part of a University of Warwick Ph.D. thesis [95]. The author noted complications from the NMR line shapes, which precluded structural characterization of tin, but the NMR data were valuable in identifying the presence of multiple oxidation states (Sn^2+^ and Sn^4+^). A similar example using ^119^Sn NMR to investigate tin fluorophosphate glasses is demonstrated in Figure 16, where wideline (static) measurements of ^119^Sn provided confirmation of mostly Sn^2+^ in these glasses, but with increasing P_2_O_5_ contents, the appearance of a Sn^4+^ resonance around −750 ppm was noted [96]. The data in Figure 16 also confirm the difficulty in using ^119^Sn NMR to study short-range structure in glasses, as the main Sn^2+^ resonance is mostly unaffected by changes in the SnF_2_:SnO ratio and, thus, the amount of Sn-O versus Sn-F bonding. Yttrium is also of marginal interest, though it might be useful as a proxy for similarly-sized rare earth elements in optically-active glasses. ^89^Y (spin-1/2; 100%) NMR in glass science has been demonstrated for a few Y-containing systems, including aluminosilicates [73] and aluminoborates [97].

### 2.3. NMR Studies of the Difficult Elements

Many elements in the Periodic Table (Figure 1) have NMR-active isotopes, are of significant interest in glass science due to various contributions to structure and properties, but are exceedingly difficult to study due to a variety of factors, similar to some of the above discussion on the “challenging” nuclei. In many cases, these nuclei suffer from small magnetic moments (i.e., low gamma) and low natural abundance of the NMR-active nuclei. Of considerable importance, at least in the context of technological glasses, is magnesium. Magnesium is a common glass modifier, but is also found in four-fold coordination and thus can be partially considered as a network former [98]. ^25^Mg (spin-5/2; 10.13%) NMR has been demonstrated in crystalline solids, but studies of glasses have been rare, and even these are complicated by a poor signal and even worse resolution. Enrichment with ^25^Mg seems to be necessary due to the low natural abundance, which as mentioned above for ^17^O NMR, precludes these types of studies in commercial or large-scale production glasses. The most comprehensive study of ^25^Mg NMR in glasses comes from the work out of Japan, where MAS and MQMAS NMR data have been analyzed [99]. In spite of many challenges, recent successes make the prospects of ^25^Mg NMR favorable [100].

Calcium (^43^Ca (spin-7/2; 0.135%)) is another important glass modifier and suffers from many of these same challenges. Successful studies of ^43^Ca using solid-state NMR have required enrichment to circumvent the low isotopic abundance, and the few natural abundance ^43^Ca NMR studies published have mainly involved crystalline materials [101,102]. There are very limited examples of ^43^Ca NMR studies of glasses, driven by recent work at a high magnetic field [103,104]. As with magnesium, the importance of this element in glass science cannot be overstated, and thus, similar advances in our ability to make NMR measurements of ^43^Ca will be forthcoming and will certainly enhance our understanding of calcium in glasses, especially when combined with powerful computational methods [105].

Nitrogen is another important anion in glass science, in particular for oxynitride glasses. However, both ^14^N (spin-1; 99.63%) and ^15^N (spin-1/2; 0.37%) NMR of these glasses are difficult, and the latter, easier isotope, requires enrichment. Limited examples of ^15^N NMR in glasses have appeared in the literature, for example, in the study of oxynitride glasses [106], but the approach has not been widely adopted, even with the current high level of interest in the mechanical and optical properties of these oxynitride glasses [107].

The other “difficult” elements that comprise this category have been studied in the solid-state, but primarily in crystalline materials. There are limited examples of ^77^Ge, ^35^S, ^93^Nb, ^125^Te, etc., in NMR studies of inorganic glasses, with ^93^Nb (spin-9/2; 100%) perhaps having the most potential at the moment [108]. ^77^Ge (spin-9/2; 9.96%) suffers from very low gamma and is quadrupolar, limiting use in NMR of glasses [109]. ^35^S (spin-3/2) has a low abundance (0.76%), but a few examples in glass NMR have been published [110], including interesting work on sulfur oxidation states in glass [111]. Tellurium, found in both oxide and chalcogenide glasses, has been detected with standard ^125^Te (spin-1/2; 6.99%) NMR methods like MAS, with the first report appearing in the early 1990s [112]. Recent studies based on ^125^Te NMR are more promising [113,114]. In fact, there are many elements that give rise to the NMR signal from glasses, but are essentially useless without more understanding of the signal and its meaning. Advances in combined computational study and NMR experiments will continue to open up new possibilities for application in glass science and ultimately will lead to continued revision of the “current” practical categorization of elements presented in Figure 1 [115]. For example, use of ^87^Sr (spin-9/2; 7.02%) NMR to study glasses, unreported during the first 50+ years of glass NMR, was recently described in a study of bioactive glasses [116].

### 2.4. Impractical and Impossible Elements in Glass NMR

The final category, which may unfortunately grow in size as the others above remain too difficult to use in the study of inorganic glasses, includes a variety of elements that do not possess isotopes with nuclear spins (e.g., argon) and/or have no bearing on glass science, or may be of interest in glasses, but are hampered by paramagnetism. The latter is unfortunately true for many transition metals and rare earth oxides, from which a variety of glass dopants is sought for their optical, electronic or other favorable attributes. On the other hand, some of these elements are used in glasses at levels higher than a dopant, for example glasses containing significant quantities of tungsten or tantalum, so the inability to fully characterize these using some of the NMR methods in this review is most unfortunate.

As with many of the challenging and important scientific problems with glasses, a limitation for study using any particular experimental or computational approach does not preclude understanding via alternate methods. This is especially true for some of the difficult elements highlighted here, and in many cases, other techniques can be leveraged to provide similar or complementary information. Even some of the most favorable NMR nuclei with widespread application in glass science, for example aluminum or boron, can be better understood using a combination of methods, and alternative approaches often add high value to something that appears to be easy.

## 3. Perspectives

Therefore, what does the future of inorganic glass NMR hold? There are continuing advances in instrumentation and methodology, driven by the need to solve complex problems in not just glass science, but in materials science and chemistry as a whole. High temperature and high pressure studies, long the purview of geochemists, are important in understanding glass melts and their properties and would require additional efforts for studies using NMR. Overall, NMR measurements, from the simple to the nearly impossible, require specialized and expensive instrumentation, as well as highly-trained researchers, to conduct these studies. This means that most glass research groups do not realistically have the resources to perform NMR studies themselves, similar to the case of using sophisticated computational or synchrotron-based methods, but certainly not as readily adopted as bench-top vibrational spectroscopies; though these need substantial expertise themselves in order to properly understand the measurements and resulting data. 

Key advances will include access to even higher magnetic fields for better sensitivity and resolution, as demonstrated by the significant advances in ^43^Ca and ^25^Mg NMR over the past few years. Faster sample spinning for MAS NMR, which now reaches upwards of 100 kHz, will improve separation of isotropic peaks from spinning sidebands for many nuclei, though the fast spinning comes with a substantial trade-off in sample volume and thus sensitivity. Additionally, a noticeable trend already evident in the field is substantial improvements in NMR methodology from young, brilliant researchers, an encouraging development for lasting improvements and the application of solid-state NMR in glass science. 

Finally, recently demonstrated as quite powerful and potentially disruptive to how we utilize NMR in glass science is the combination of experiment and computational methods, offering the ability to further extract structural details from easily-acquired NMR data and enabling a better understanding of less common elements and the inherent distribution of structural features common to inorganic glasses. There are several excellent examples of density functional theory calculations of important NMR-related parameters in glasses. Some of these involve systems for which we already have considerable knowledge, for example the work on silicate and aluminosilicate glasses, but where calculations and NMR experiments both contribute to a better understanding of network polymerization and distributions between different network-forming polyhedra [117]. Chemical shielding and quadrupolar coupling parameters have been calculated for a tectosilicate calcium aluminosilicate, similar to compositions used in the examples of Figure 6, and then used to simulate MAS and 3QMAS NMR spectra for ^27^Al and ^17^O. Good agreement with experimental NMR data then allowed these researchers to use the results from MD simulations to describe in even greater detail the short- and intermediate-range structure [118]. Other similar studies have leveraged this advanced structural understanding to further correlate NMR parameter distributions with short-range structural features, providing valuable insight into chemical and topological disorder [119]. As evidenced by these few examples of combined computational and experimental studies, there have been tremendous advances in our understanding of glass structure, and such developments will continue to advance research on glasses.
